# Aquatic Bacterial Diversity, Community Composition and Assembly in the Semi-Arid Inner Mongolia Plateau: Combined Effects of Salinity and Nutrient Levels

**DOI:** 10.3390/microorganisms9020208

**Published:** 2021-01-20

**Authors:** Xiangming Tang, Guijuan Xie, Keqiang Shao, Wei Tian, Guang Gao, Boqiang Qin

**Affiliations:** 1 Taihu Laboratory for Lake Ecosystem Research, State Key Laboratory of Lake Science and Environment, Nanjing Institute of Geography and Limnology, Chinese Academy of Sciences, Nanjing 210008, China; xieguijuan18@mails.ucas.ac.cn (G.X.); kqshao@niglas.ac.cn (K.S.); tianwei94glas@163.com (W.T.); guanggao@niglas.ac.cn (G.G.); qinbq@niglas.ac.cn (B.Q.); 2 College of Resources and Environment, University of Chinese Academy of Sciences, Beijing 100049, China

**Keywords:** bacterial diversity, bacterial community composition, community assembly, salinity, eutrophication, environmental filtering

## Abstract

Due to the recent decades of climate change and intensive human activities, endorheic lakes are threatened by both salinization and eutrophication. However, knowledge of the aquatic bacterial community’s response to simultaneous increasing salinity and trophic status is still poor. To address this knowledge gap, we collected 40 surface water samples from five lakes and six rivers on the semi-arid Inner Mongolia Plateau, and investigated their bacterial communities using 16S rRNA gene-targeted amplicon sequencing. We found that bacterial species diversity significantly decreased from the mesotrophic freshwater river habitat to the eutrophic high-brackish lake habitat; salinity was more important than trophic status in explaining this decreased diversity. Salinity was the most important environmental factor in shaping community composition, while increased nitrogen loading was more important in structuring predicted functional composition. Within the lake habitats, the impact of environmental filtering on bacterial community assembly increased with the increasing salinity. The results suggested that the elevated salinity and nutrients have combined effects on the aquatic bacterial community, resulting in dramatic declines in species diversity, and promoted the importance of deterministic processes in community assembly. Our findings provide new insights into bacterial communities’ responses to the intensified climate-driven and anthropogenic environmental changes in aquatic ecosystems.

## 1. Introduction

Heterotrophic bacteria are key decomposers in aquatic ecosystems, while autotrophic bacteria (such as *Cyanobacteria*) are key producers in eutrophic waterbodies. They play a crucial role in nutrient cycling and organic matter transformation [1,2]. In recent decades, natural aquatic ecosystems have experienced increasing salinization and eutrophication due to climate change and intense human activities [3,4,5]. Thus, the understanding of how bacterial communities respond to increasing lake salinization and eutrophication is important as we are in the Anthropocene [6,7].

Over the past two decades, many studies have been carried out on bacterial diversity and community composition in lakes with different nutrient levels [8,9,10]. Although the relationship between bacterial diversity and trophic status is complicated, it has been suggested that the maximum diversity occurs at intermediate levels of nutrients or productivity [10,11].

Salinity is an additional major environmental factor that shapes bacterial communities [12,13,14]. The effects of salinity on bacterial diversity are varying, with studies reporting a negative correlation [13,15,16], no effect [14,17], or a peak diversity at low salinity [18]. However, no research has investigated how bacterial diversity and bacterial community composition (BCC) respond to elevated nutrients and increased salinity simultaneously.

Increasing environmental pressure, including salinization and eutrophication, can affect bacterial community assembly mechanisms at both the regional and local scales. Disentangling the underlying mechanisms driving bacterial community assembly is a central challenge in microbial ecology. It is accepted that community assembly is affected by the trade-off between stochastic and deterministic processes [19]. If communities are stochastically assembled, their composition is mainly determined by dispersal limitation (large spatial scales), mass effects/homogenizing dispersal (in the case of strong connected systems), or ecological drift. In contrast, deterministically assembled communities arise due to selection by local environmental filtering or biotic interactions [20,21,22]. However, the relative contributions of stochastic and deterministic processes in shaping aquatic community assembly remain poorly understood. This knowledge gap limits our capacity to predict the responses of bacterial communities’ structures and functions to changes in their surrounding environment.

Due to climate warming and inappropriate anthropic activities, lakes in the semi-arid Inner Mongolia Plateau have been increasingly threatened by salinization and eutrophication in recent decades [23,24,25]. Increasing air temperature and evaporation, decreasing precipitation, and the intensification of agriculture, over-grazing and fish-farming are thought to be the main drivers of the shrinking, and subsequent salinization and eutrophication, of most endorheic lakes in this region [25,26,27].

To explore the combined impacts of salinity and nutrient levels on bacterial communities, we studied the bacterial communities in both separated and connected lakes, and their connected rivers, with different levels of salinity and nutrients in the semi-arid Inner Mongolia Plateau. We hypothesized the following: (1) bacterial diversity would decrease as salinity and nutrients increased; (2) differences in salinity and nutrient levels would be reflected in BCC and community function; and (3) the importance of the deterministic community assembly process (relative to stochastic processes) would increase with salinity and nutrient levels.

## 2. Materials and Methods

### 2.1. Study Area and Sample Collection

**Figure 1:**
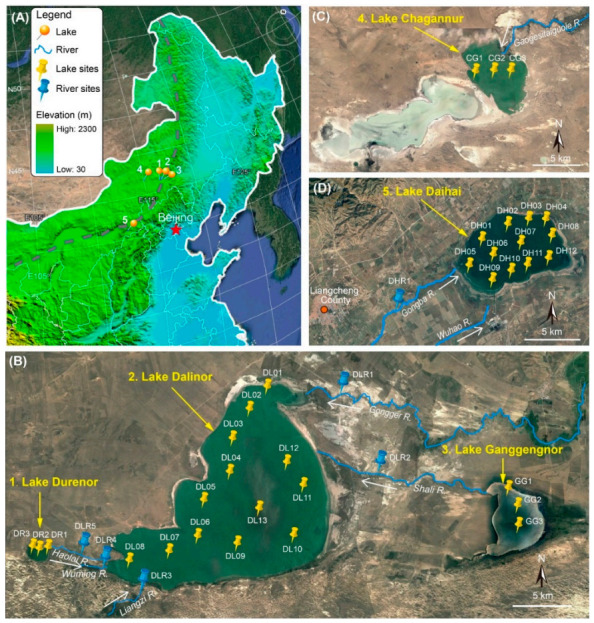
Satellite images of the study sites in the Inner Mongolian Plateau of China (from http://www.maps.google.com). (**A**) Northeast China, showing the location of the five lakes. Grey dashed line shows the current northern limit of the East Asian summer monsoon. (**B**) Lake Dalinor basin showing the connected lakes and the 19 sampling sites in the three lakes, and 5 sampling sites among the five rivers. (**C**) Lake Chagannur showing three sampling sites. (**D**) Lake Daihai showing 12 sampling sites in the lake, and 1 sampling site in one of its upstream rivers. White arrows show the waterflow directions of the rivers.

We chose five lakes in the Inner Mongolia Plateau with salinity levels from freshwater to high-brackish, and trophic status from light eutrophic to hyper eutrophic (Table 1). These lakes are located at the transition margin of the semi-humid to semi-arid areas of the middle temperate zone of China, with the furthest distance of about 470 km (Figure 1). The climate is influenced by the East Asian summer monsoon [28], a steady flow of warm, moist air from the tropical oceans, which largely controls the position and extent of the monsoonal rainfall belt in northern China.

Three of our studied lakes (Durenor, Dalinor and Ganggengnor) lie within the 5550 km^2^ catchment of the Dalinor Basin (Figure 1B), a typical inland lake basin [24]. The largest lake, Lake Dalinor (43°13′–43°23′ N, 116°29′–116°45′ E), a low-brackish lake, receives its water from two permanent rivers from the northeast, and three intermittent rivers from the southwest. The major inflow of this lake is the Gongger River, which supplies about 75% of the total discharge. The freshwater Lake Ganggengnor (43°14′–43°18′ N, 116°53′–116°57′ E) and Lake Durenor (43°14′–43°15′ N, 116°24′–116°25′ E) are located at the east and west of Lake Dalinor, respectively, and both flow into Lake Dalinor [24].

Another two endorheic lakes lie outside of the Dalinor Basin. Lake Chagannur (43°22′–43°29′ N, 114°46′–115°03′ E) lies approximately 130 km west from Lake Dalinor (Figure 1C). Lake Daihai (40°32′–40°37′ N, 112°36′–112°46′ E) is a hydrologically closed, high-brackish lake, with a catchment of 2289 km^2^, and is supplied by two permanent rivers from the southeast (Figure 1D).

We also collected six water samples from six rivers. Five of the rivers flow into Lake Dalinor, including one river (Haolai River) that connects Lake Durenor and Lake Dalinor, and another river (Shali River) connecting Lake Ganggengnor and Lake Dalinor (Figure 1B). Another sample was collected from Gongba River, which supplies water for Lake Daihai (Figure 1D).

Between 8 September and 14 September 2018, a total of 40 water samples were collected from 34 lake sites (Durenor 3, Dalinor 13, Ganggengnor 3, Chagannur 3, Daihai 12) and 6 river sites (Figure 1). At each site, 10 L surface water (top 50 cm) was collected, and subsamples of 150–300 mL for molecular analysis were filtered through a 0.2 μm pore-size polycarbonate filter (Isopore^TM^, Millipore, Dublin, Ireland), using a hand-driven vacuum pump in the field. During transportation back to the laboratory, the filters were stored at −20 °C in a vehicle-mounted refrigerator; subsequently they were stored at −80 °C in the laboratory for DNA extraction. The remaining water samples were transported to the laboratory for immediate chemical analysis.

### 2.2. Measuring Environmental Parameters

At each of the 40 sites, we measured seven physicochemical parameters in situ using a multiparameter water quality sonde (YSI EXO2, Yellow Springs Instruments Inc., Yellow Spring, OH, USA) at approximately 30 cm depth: water temperature (WT, °C), pH, dissolved oxygen (DO), electrical conductivity (EC), salinity, total dissolved solids (TDS) and fluorescent dissolved organic matter (fDOM). The transparency (SD) of lake sampling sites was measured using a Secchi disk.

A further 11 parameters were measured upon return to the laboratory, according to standard methods [31]: total nitrogen (TN), total dissolved nitrogen (TDN), ammonia nitrogen (NH_4_), nitrate (NO_3_), total phosphorus (TP), total dissolved phosphorus (TDP), orthophosphate (PO_4_), suspended solids (SS), loss on ignition (LOI), chemical oxygen demand (COD) and chlorophyll-a (Chl-*a*).

Based on ecosystem types and the salinity, the samples were assigned to four habitat groups: freshwater rivers (Group I), freshwater lakes (Lakes Durenor, Ganggengnor and Chagannur, Group II), low-brackish Lake Dalinor (Group III), and high-brackish Lake Daihai (Group IV).

### 2.3. Bacterial Diversity and Community Composition

The DNA from each water sample was extracted using FastDNA^®^ Spin Kit for Soil (MP Biomedicals, Solon, OH, USA) according to the manufacturer’s instructions. The V3–V4 hypervariable regions of the bacterial 16S rRNA genes were amplified using the primer set 338F (5′-ACTCCTACGGGAGGCAGCAG-3′) and 806R (5′-GGACTACHVGGGTWTCTAAT-3′) [32]. The polymerase chain reaction (PCR) mixtures contained 20 ng diluted DNA template, 0.4 μM of each primer, and Phusion^®^ high-fidelity PCR master mix (New England Biolabs Inc., Ipswich, MA, USA). The PCR program was as follows: 3 min of initial denaturation at 98 °C followed by 30 cycles of denaturation at 98 °C (45 s), annealing at 55 °C (45 s), elongation at 72 °C (45 s), and a final extension at 72 °C for 7 min. Following the purification of the amplicon pools using AMPure XP beads (Beckman Coulter, Indianapolis, IN, USA), sequencing was performed on the Illumina MiSeq PE300 platform by the Beijing Genomics Institute (BGI), China, using a pair-end sequencing (2 × 300 bp) strategy.

The bioinformatic analysis was carried out on CLC Genomics Workbench 20.0 (Qiagen, Aarhus C, Denmark) with the Microbial Genomics Module, according to the tutorial “OTU Clustering Using Workflows (https://resources.qiagenbioinformatics.com/tutorials/OTU_Clustering_Workflows.pdf)”. After importing raw reads, a standard quality control process was carried out, including merger paired reads (minimum overlap of 200 bp), trim off adapters and the primer sequences, and quality trimming (trim using quality scores with a limit of 0.05; trim ambiguous nucleotides with a maximum number of ambiguities of 2) [33].

Bacterial phylotypes were identified and assigned to operational taxonomic units (OTUs, 97% similarity). To minimize the random sequencing error, we filtered out low-abundance OTUs (<10 reads) from the OTU table. Taxonomic classification was performed by comparing reads against the SILVA small subunit rRNA (SSU) database v132 at the 80% confidence level [34]. Sequences associated with chloroplast and chimeras were excluded from subsequent analysis. Bacterial α-diversity was calculated after normalizing the sequencing depth, based on the sample with the smallest sequencing effort (17,629 reads in this study). To evaluate β-diversity (compositional variation from site to site) among different groups, we performed cluster analysis based on the Bray–Curtis distance.

### 2.4. Functional Annotation of Bacterial Communities

The annotation of bacterial functional composition (BFC) was performed by using the database FAPROTAX on the normalized OTU table [35]. Each taxonomically annotated OTU was compared automatically against the FAPROTAX_1.1 database (including 7820 annotations and covering 4724 taxa) using a web-based platform (http://www.ehbio.com/ImageGP/index.php/Home/Index/FAPROTAX.html).

### 2.5. Environmental and Spatial Factors Associated with Patterns of BCC and BFC

To explore the significant environmental variable combinations that were associated with BCCs and the predicted functional profiles, we performed redundancy analysis (RDA) using the vegan package in R [36]. To reduce the effect of highly abundant OTUs, the OTUs data were Hellinger-transformed for all downstream analysis [37]. To eliminate the different measurement scales, environmental (explanatory) variables were normalized. Then, a forward selection procedure was performed using a Monte Carlo test with 999 permutations. Only variables that explained a significant (*p* < 0.05) additional proportion of total variance were included in the subsequent selection. Finally, the variance inflation factors (VIF) of each selected significant variable were calculated, and the variables with VIF values above 10 (indicating strong collinearity) were removed from the model. The distance–decay model was fitted with spatial distance (calculated by geographical coordinates using *SoDA* v1.0-6 package in R) and the bacterial community Bray–Curtis similarity among samples [38].

Based on the results of RDA and distance–decay analysis, a variation partitioning approach (VPA) was used to test the relative importance of environmental variables and spatial factors in structuring bacterial communities. The roles of spatial factors were estimated by the principal coordinates of neighbor matrices (PCNM) method [39]. A forward selection procedure [40] was performed to select significant linear trend factors. Then, the variation in the BCCs was partitioned between the selected significant environmental variables, the extracted PCNM spatial variables, and the linear trend factors, using a partial redundancy analysis (pRDA) in the vegan package [41]. This pRDA allows the total explanations of BCC variation to be decomposed into fractions that indicate the relative importance of pure environmental variables, pure spatial variables, spatially structured environmental variation (shared fraction), and unexplained variation.

### 2.6. Ecological Processes Govern the Microbial Community Assembly

To assess the relative importance of the deterministic and stochastic processes driving bacterial community assembly, the null model analysis using abundance-based β-diversity matrices [42,43,44] was performed using the R code described by Zhang et al. [45]. To quantitatively estimate the strength of the stochastic ratio in shaping microbial community variation, we used the difference between the observed similarity matrices and the null model expectation. The significance of the *p*-value was calculated by comparing the observed F value with those from 1000 randomized data sets using permutational multivariate analysis of variance (PERMANOVA).

### 2.7. Statistical Analyses

The statistical analyses and the visualization were performed using R 3.5.3 (https://www.r-project.org) and the RStudio 1.1.463 interface, unless otherwise indicated. Data visualization was performed using the packages “*ggplot2*”, “*pheatmap*” and “*ggcor*” in R.

The predicted BFC from FAPROTAX annotation, based on raw OTU tables, was compared among different groups using cluster analysis and the two-sided White’s non-parametric *t*-test with Benjamini–Hochberg FDR. Significance was assigned when the *p*-value < 0.05 and LDA > 3.5. The comparisons were visualized on the software STAMP v2.1.3 [46].

To statistically test the difference of BCCs among the four habitat groups, we performed analysis of similarity (ANOSIM) and PERMANOVA with 999 permutations [47]. The ANOSIM then generated a test statistic *r*, with a score of 1 indicating complete separation, and 0 indicating no separation.

The Mantel permutation test was performed to calculate the Spearman’s rank correlations among dissimilarity entries of BCCs (Bray–Curtis distance), relative functional composition (Bray–Curtis distance), geographic distance (Euclidean distance), and the scaled environmental matrices (Euclidean distance), using the *vegan* and *ggcor* packages in R with 9999 permutations [36].

## 3. Results

### 3.1. Environmental Characterization

**Figure 2:**
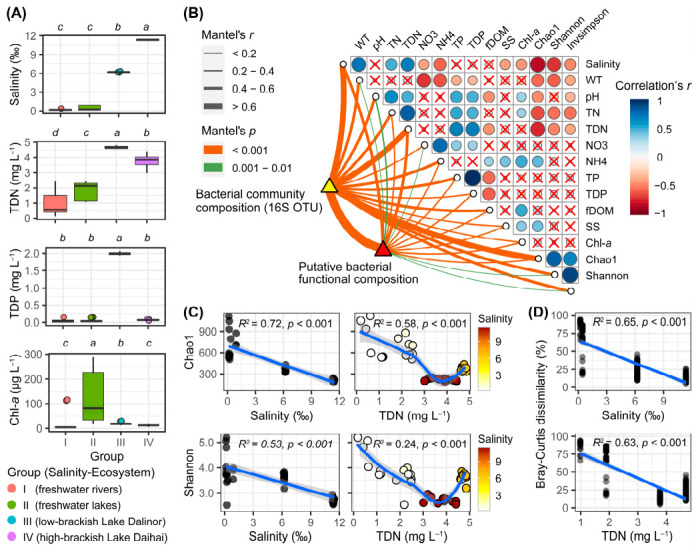
Main environmental parameters, and the diversity of bacterial communities, among the four habitat groups based on salinity gradients and ecosystem types. (**A**) Boxplot comparison of the four main environmental parameters among the four groups, using Kruskal–Wallis test to examine the significant levels of the differences. Above each panel, the different lower-case letters indicate significant differences (*p* < 0.05) among different groups. TDN, total dissolved nitrogen; TDP, total dissolved phosphorus; Chl-*a*, chlorophyll-*a*. (**B**) Pearson’s correlation coefficients of the main 12 environmental factors, the three bacterial diversity indices (Chao1, Shannon, Invsimpson), bacterial community composition, and predicted bacterial functional composition using Mantel tests. Circle size corresponds to the value of the correlation coefficients. Circles with a cross denote the statistically non-significant pairwise correlations. Line width corresponds to the Mantel’s r statistic for the corresponding distance correlations, and line color denotes the statistical significance based on 9999 permutations. (**C**) Regression of bacterial α-diversity indices Chao1 and Shannon using salinity and TDN. The linear or LOESS fits were performed with the solid blue lines, while the grey shaded areas represent 95% confidential intervals. The adjusted *R*^2^ and significance *p*-values are presented in each panel using a linear model. In the right panels, the color gradients represent salinity. (**D**) Regression of bacterial β-diversity (Bray–Curtis dissimilarity) using salinity and TDN. Each circle represents the Bray–Curtis dissimilarity values between two samples in each habitat group, using the average values of salinity and TDN in each habitat group as the values in *x*-axis.

Among the 40 samples, there was a distinct salinity gradient (Figure 2A). In the six river samples (Group I), salinity ranged from 0.14‰ to 0.34‰ with a mean of 0.21‰; in the nine freshwater lake samples (Group II), salinity ranged from 0.16‰ to 0.92‰ with a mean of 0.44‰; in the two brackish lakes, Dalinor and Daihai, the mean and standard deviation of salinity were 6.22 ± 0.03‰ and 11.36 ± 0.06‰, respectively. Correlation analysis showed that salinity was positively correlated with TDN (*r* = 0.70, *p* < 0.001) (Figure 2B).

The low-brackish Lake Dalinor (Group III) had the highest concentrations of TDN and TDP among the four groups, while the freshwater lakes (Group II) had significantly higher concentrations of Chl-*a* than that in the other groups (Figure 2A). Data on the other 15 environmental parameters for each group are presented in Supplementary Figure S1. The highest concentrations of TN, TP and Chl-*a* were ~5.88 mg L^−1^ (Lake Ganggengnor), 2.11 mg L^−1^ (Lake Dalinor) and 288 μg L^−1^ (Lake Ganggengnor), respectively.

### 3.2. Bacterial α- and β-Diversity

We generated 1,300,863 high-quality reads with a mean of 32,521 reads per sample. Across the 40 samples, the reads were classified into 1902 OTUs. The rarefaction curves of Chao1 bias-corrected richness approached an asymptote after 15,000 reads (Figure S2), indicating enough sequencing depth for further analysis.

The numbers of OTUs in Groups I–IV were 1467, 1134, 509 and 305, respectively (Figure S3A). Only 8% (91) of the total OTUs were shared by all groups. Within freshwater rivers (Group I), 35% OTUs were exclusive to this habitat. Within lake habitats, the proportions of exclusive OTUs increased with increasing salinity, from 13% in freshwater (Group II), to 16% in low-brackish (Group III), and to 18% in high-brackish (Group IV) groups.

Alpha-diversity analysis revealed a significantly decrease in species richness and Chao1 indices from freshwater river habitats to the high-brackish Lake Daihai (Figure S3B). The Bray–Curtis dissimilarity in BCC within each group (from Group I to IV), expressed as the mean ± SD, was 85.6 ± 8.7%, 64.0 ± 24.4%, 21.5 ± 8.8% and 13.0 ± 4.2%, respectively (Figure S3C), representing a decrease in β-diversity from Group I to Group IV.

Our correlation analysis showed that the bacterial α-diversity indices were negatively correlated with salinity and TDN (Figure 2B). Regression analysis showed that both bacterial α-diversity (Chao1 and Shannon) and β-diversity decreased significantly with the increasing salinity and TDN (*p* < 0.001; Figure 2C,D).

### 3.3. Bacterial Taxonomy and Community Structure

**Figure 3:**
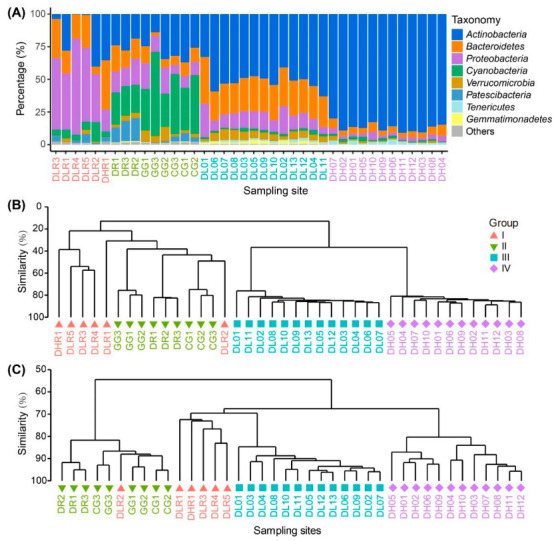
Taxonomic composition, bacterial community composition (BCC) and predicted bacterial functional composition (BFC) among the four habitat groups based on salinity gradients and ecosystem types. (**A**) Taxonomy of the bacteria at the phylum level. (**B**) Cluster analysis dendrogram of the BCC. (**C**) Cluster analysis dendrogram of predicted BFC. Samples are those from freshwater rivers (Group I), freshwater lakes (Group II), low-brackish Lake Dalinor (Group III), and high-brackish Lake Daihai (Group IV).

In the freshwater river habitats, the dominant phyla were *Proteobacteria*, *Bacteroidetes* and *Actinobacteria*, accounting for 47%, 22% and 18% of the total reads, respectively (Figure 3A). In contrast, in the freshwater lake habitats, the dominant phyla were *Cyanobacteria*, *Actinobacteria* and *Proteobacteria*, accounting for 35%, 26% and 14% of the total reads. In brackish Lake Dalinor and Lake Daihai, the dominant phyla were *Actinobacteria*, *Bacteroidetes* and *Proteobacteria*, which respectively accounted for 51%, 26% and 13% of the total reads in Lake Dalinor, and for 87%, 5% and 3% of the total reads in Lake Daihai.

At the genus level, in the freshwater river and lake habitats, the taxonomic composition varied considerably among different sampling sites, whereas the taxonomic composition was similar among the sampling sites in Lake Dalinor and Lake Daihai (Figure S4). The most dominant genus in Group I–IV was *Flavobacterium*, hgcI clade, CL500-29 and *Rhodoluna*, respectively.

Cluster analysis showed a distinct separation of the BCCs from freshwater habitats to brackish habitats (Figure 3B). Both PERMANOVA analysis and ANOSIM (Table S1) showed that the differences in BCCs among the four groups were all statistically significant (*p* ≤ 0.0002).

**Figure 4:**
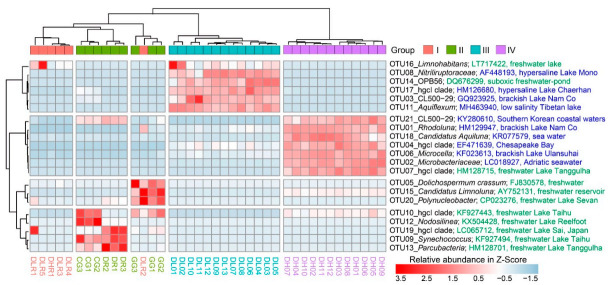
Heatmap of the 21 most abundant OTUs (mean relative abundance > 1%; descending order from OTU01 to OTU21 in abundance) among freshwater rivers (Group I), freshwater lakes (Group II), low-brackish Lake Dalinor (Group III), and high-brackish Lake Daihai (Group IV). The most related counterpart of each OTU was compared using BLAST in the database of the National Center for Biotechnology Information (NCBI). The NCBI access number, and the habitat from which the organism was isolated, are given after the taxonomic information and the OTU number. Information in green denotes that the clones were isolated from freshwater, and information in blue denotes that the clones were isolated from brackish or marine habitats.

Differential abundance analysis revealed that the 21 most abundant OTUs (mean relative abundance > 1%) were significantly different (*p* < 0.001) among the four groups (Figure 4). From Group I to Group IV, there was a clear pattern of distinct enrichment of specific OTUs in a certain habitat. For example, cyanobacterial OTUs (5, 9 and 12) had higher relative abundances in freshwater lakes, while the actinobacterial OTUs (1, 2, 4, 6, 18 and 21), which had higher relative abundances in the high-brackish Lake Daihai, were closely related to those found in other brackish lakes or coastal sea water.

### 3.4. Bacterial Functional Composition

The functional annotation of OTUs revealed a rich repertoire of metabolic functional types. In total, 646 out of 1902 OTUs (34%) were assigned to at least one functional type, representing 55 out of 90 functional types in the functional annotation of the FAPROTAX 1.1 database (Figure S5). The cluster analysis of the functional profiles showed distinct separation among the four habitat groups, except for the sample DLR2 (Figure 3C). Water collected from this site (in Shalin River) came from the hyper eutrophic Lake Ganggengnor (Figure 1). This may be the reason why sample DLR2 had distinct differences in nutrients, bacterial community and functional compositions compared with other river samples. Both the PERMANOVA analysis and ANOSIM (Table S1) showed that the differences in predicted functional composition among the four groups were all statistically significant (*p* ≤ 0.0004).

A total of 43 functional types were significantly differently distributed among the four habitat groups. Among the functional types, photoautotrophy (contributed by *Cyanobacteria*) and methanotrophy (contributed by the families of *Beijerinckiaceae* in *α-Proteobacteria* and *Methylococcaceae* in *β-Proteobacteria*) were significantly enriched in the freshwater river habitat, while chemoheterotrophy was significantly enriched in the other three groups (Figure S6A). Methylotrophy (contributed by *α*-, *β*-, and *γ-Proteobacteria*) was significantly enriched in Lake Dalinor, and fermentation was significantly enriched in Lake Daihai. Nitrogen fixation was significantly enriched in the freshwater river habitat, while nitrogen respiration and denitrification were significantly enriched in Lake Dalinor (Figure S6B).

### 3.5. Environmental Drivers on BCC and BFC

The Mantel test showed that BCC was moderately correlated with both salinity (*r* = 0.68, *p* < 0.001) and TDN (*r* = 0.64, *p* < 0.001) (Figure 2B). The predicted BFC was correlated with TDN (*r* = 0.47, *p* < 0.001) and salinity (*r* = 0.41, *p* < 0.001). In addition, BCC and BFC were also significantly correlated with each other (*r* = 0.72, *p* < 0.001).

**Figure 5:**
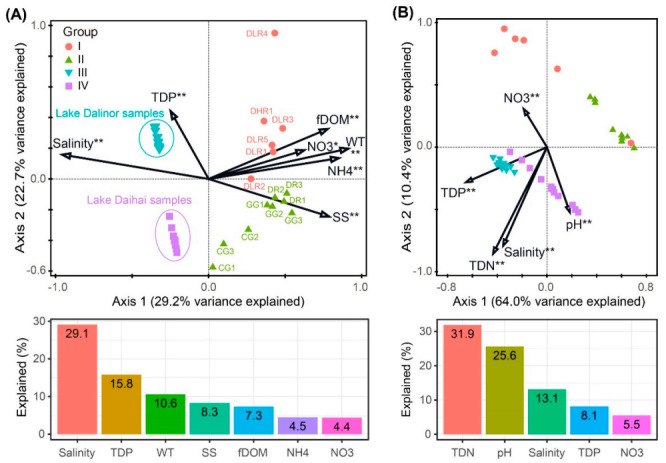
Redundancy analyses (RDA) ordination plots showing the significant environmental factors in structuring variations in (**A**) bacterial community composition, and (**B**) predicted bacterial functional composition. Bar plots are presented below each RDA plot panel, showing the variation explained by each factor. Significance levels: * corrected *p*-value < 0.05, ** corrected *p*-value < 0.01. TDP, total dissolved phosphorus; WT, water temperature; SS, suspended solids; fDOM, fluorescent dissolved organic matter; NH4, ammonia nitrogen; NO3, nitrate; TDN, total dissolved nitrogen.

The forward selection procedure in RDA revealed that the variation in BCC was significantly explained by seven environmental factors: salinity, TDP, WT, SS, fDOM, NH4 and NO3 (Figure 5A). These factors explained 75.7% of the variation in BCC. Among them, salinity was the most important factor, itself explaining 29.1% of the total variation.

The variation in bacterial functional profiles was significantly explained by five environmental factors: TDN, pH, salinity, TDP and NO_3_. These factors explained 81.7% of the variation in BFC (Figure 5B). Among these five environmental variables, TDN was the most important factor, which alone accounted for 31.9% of the total functional variation.

### 3.6. Contributions of Geographic and Environmental Factors to BCC

Our results showed that the similarity in BCC decreased with increasing geographic distance (*p* < 0.001; Figure S7). However, comparisons from within each sampling group showed that the distance–decay pattern was only significant in the freshwater lake ecosystem and in the low-brackish Lake Dalinor (*p* < 0.001), but not in the other two habitats (*p* > 0.05).

**Figure 6:**
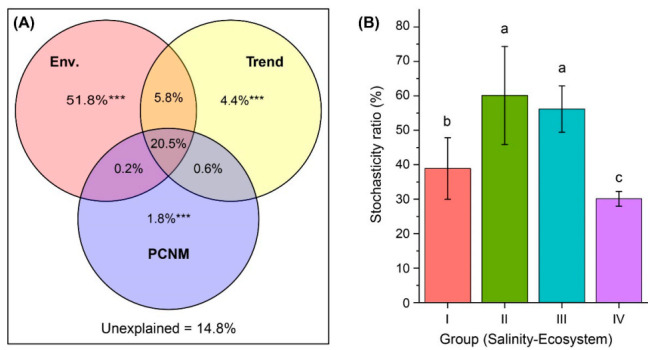
Drivers of bacterial community composition (BCC) and assembly. (**A**) Venn diagram presenting the partitioning results for the variations in BCC by environmental variables (Env.), and the spatial variables of linear trend and principal coordinates of neighbor matrices (PCNM). The fraction values displayed are computed from adjusted R-squares. *** Permutation test *p*-value = 0.001. (**B**) Relative importance of stochastic mechanism in community assembly in freshwater rivers (Group I, mean salinity = 0.21‰), freshwater lakes (Group II, mean salinity = 0.44‰), low-brackish Lake Dalinor (Group III, mean salinity = 6.22‰), and high-brackish Lake Daihai (Group IV, mean salinity = 11.36‰). Different lower-case letters indicate significant differences (*p* < 0.01) among groups.

Our PCNM analysis showed that 85.2% of the variation in BCCs was explained by environmental factors, linear trend and spatial scale (Figure 6A). The variation partitioning results revealed that the three sources of variation make unequal but significant (*p* = 0.001) unique contributions: the environment variation was the largest (51.8%), while the trend alone (4.4%) and the spatial variation played smaller roles (1.8%). Moreover, 20.5% of the variation was explained jointly by environmental and spatial variables.

### 3.7. Influence of Stochastic and Deterministic Processes on Bacterial Community Assembly

The null model analysis and PERMANOVA test showed that the observed similarity of the actual community of each sampling group was significantly distinguishable (*p* < 0.001) from that of the null expectation. The influence of the stochastic ratio (SR) on community assembly in the freshwater river habitat (38.9%) was significantly lower than that in the freshwater lake habitat (60.1%) (Figure 6B). Within the lake habitat, the SR decreased with increasing salinity. In Lake Daihai, with the highest salinity, the SR was the lowest (30.1%), suggesting that deterministic processes play more important roles in shaping lake bacterial communities as salinity increases.

## 4. Discussion

### 4.1. Elevated Salinity and Nutrient Levels Decreased Bacterial Species Diversity

In this study, we observed a significant decrease in bacterial species diversity in aquatic habitats with increasing salinity and nutrients (Figure 2C and Figure S3B). Salinity was a more powerful factor in decreasing bacterial species diversity than trophic status was (Figure 2C). Our observation of the relationship between species diversity and salinity is consistent with the finding of Yang et al. [16], who found that the microbial diversity indices in surface sediments of the Qinghai-Tibetan lakes decreased with increasing salinity. However, our results contrast with the findings of Ji et al. [13], who studied bacterial diversity in Tibetan Plateau lakes, and found that brackish lakes with salinity of 3‰ to 20‰ harbored bacterial diversities similar to, or even higher than, freshwater lakes. This discrepancy may be attributed to the different trophic status of the studied lakes: eutrophic in our present study, and oligo- to mesotrophic in the study by Ji et al. [13].

In the present study, the significant loss of bacterial diversity from freshwater river habitats to the high-brackish Lake Daihai could be due to the dual pressures of salinity and nutrients. Using denaturing gradient gel electrophoresis (DGGE), Wang et al. [18] reported that although the bacterial richness in Tibetan lakes increased at salinities lower than 1‰, there was no obvious decrease at higher salinities. They attributed the peak of bacterial diversity in salinity around 1‰ to higher nutrient levels at this salinity. However, in other studies using high-throughput sequencing, bacterial diversity significantly negatively correlated with salinity, due to the salt-induced osmotic stress, which would eliminate the salt-sensitive bacteria and thus decrease the species diversity [16,48].

Nutrient availability plays an important role in bacterial diversity [49]. Although inorganic nitrogen (N) and phosphorus (P) are essential for bacterial growth, their enrichment could decrease bacterial diversity, in both soil [50,51] and aquatic ecosystems [8,52]. Maximum bacterial diversity generally occurs at intermediate levels of nutrients or productivity [10,11]. Within the lake ecosystem we investigated, the Chao1 richness in the high-brackish, hyper trophic (average TDN = 3.78 mg L^−1^) Lake Daihai was only about one-third of that in the freshwater lakes (average TDN = 1.89 mg L^−1^). In addition, we found significantly lower species diversity in the freshwater lakes than in the freshwater rivers with similar salinity, but much lower concentrations of TDN. In addition to the different ecosystem types (river vs. lake), different nutrient levels may partially account for the loss of species diversity.

### 4.2. Contrast Relative Importance of Salinity and Nutrient for BCC and BFC

Our data support the second hypothesis that both salinity and nutrient levels have strong impacts on BCC and on predicted BFC (Figure 3 and Figure 4). What is novel about our result was that salinity played a more important role in the variations in BCCs than did nutrients, while nutrients played a more important role in the variations in BFC than did salinity (Figure 5).

Our findings on the importance of salinity in shaping BCCs agree with previous reports for the Qinghai-Tibetan lakes [13,16,48], Lake Bosten in northwestern China [53,54], and coastal Antarctic lakes [55]. On a global scale, Lozupone and Knight [12] reported that salinity was the major determinant of BCC in natural environments. Although salinity can provide a physiological barrier for certain bacterial groups [56,57,58], other bacterial groups may tolerate high salinity and become very abundant [14]. In our present study, we characterized the taxonomic divergence of bacterial groups along the salinity gradient (Figure 3A and Figure 4). For example, elevated salinity increased the relative abundance of Actinobacteria (from 18% in freshwater rivers to 87% in the high-brackish Lake Daihai), but decreased the abundance of Proteobacteria (from 47% in freshwater rivers to 3% in Lake Daihai) (Figure 3A). The heatmap of the top 21 OTUs also showed a clear succession along salinity, with most of the abundant OTUs (6/7) in Lake Daihai having their closest counterparts being from brackish habitats (Figure 4). Although the succession may also have been related to nutrient levels, salinity was the major determinant of taxonomic succession.

We found that the major environmental determinant of BFC was TDN rather than salinity (Figure 5B). Our results agree with Berga et al. [59], who found that bacterial communities were functionally resistant to salinity levels as high as 12‰. Compared with freshwater rivers, the elevated TDN in freshwater lakes favors the dense growths of *Cyanobacteria* (Figure 3A and Figure S4), which could explain the significantly enhanced photoautotrophy in this habitat compared to the other three habitats (Figure S6A). Significantly enhanced nitrogen respiration and denitrification were found in Lake Dalinor, with the highest concentrations of TDN (4.68 mg L^−1^), while nitrogen fixation was significantly enhanced in freshwater rivers, with the lowest concentrations of TDN (0.99 mg L^−1^) (Figure S6B). This finding is consistent with the recent study of a highly polluted river by Yang et al. [60], which showed that the functional groups related to nitrogen metabolism were most abundant when nitrogen was most enriched. It appears that aquatic ecosystems can attenuate the negative impacts of nutrient enrichment via bacterial community functional adaptation.

### 4.3. Importance of Salinity-Induced Environmental Filtering in Bacterial Assembly

Within the four habitat groups, we found that the relative importance of stochastic processes in freshwater and low-brackish lake habitats (Group II and Group III) was significantly higher than in river habitats and in the high-brackish Lake Daihai (Group I and Group IV, Figure 6B). This result was supported by the results of distance–decay analysis, because the biogeographic patterns (a kind of stochastic effect) have only been observed in the freshwater lake habitats and in low-brackish Lake Dalinor (Figure S7). The low level of similarity in BCCs among the three freshwater lakes (Figure 3B) suggested that dispersal limitation has a strong impact on bacterial community assembly in the inland separated freshwater lakes.

A significantly lower importance of stochastic processes was found in the middle eutrophic, high-brackish Lake Daihai (Group IV) compared with the hyper eutrophic, low-brackish Lake Dalinor (Group III) (Figure 6B), which indicated that salinity, rather than nutrient level, is the important deterministic factor in bacterial community assembly. Thus, our third hypothesis was partially supported.

Overall, our results revealed that salinity was the most important factor in environmental filtering (a deterministic process) in relation to bacterial community assembly in high-brackish lake ecosystems. This result is consistent with recent findings in Tibetan lakes [48]. In the high-brackish lake habitat, the strong selection stress imposed by the increased salinity may provide a constant and buffered environment, which, when coupled with the high population growth rates of bacteria [22], favors the thriving of an autochthonous dominant community adapted to this high salinity niche.

A significantly lower importance of stochastic processes was also found in freshwater river habitats (Group I) compared with the freshwater lake habitat (Group II) and the low-brackish lake habitat (Group III) (Figure 6B). However, lesser stochasticity ratios are estimated in the freshwater river and high-saline lake habitats (Groups I and IV), which may indicate that different drivers apply to the system to increase determinism. Higher environmental heterogeneity in rivers may supply more ecological niches for riverine bacteria, thus increasing the relative importance of the deterministic effect, which is different with salinity-induced environmental filtering, resulting in the increasing importance of the deterministic effect in the high-saline lake habitat.

It is possible that our findings may be somewhat limited by the gradients of salinity and nutrient levels in the sampled rivers and lakes. More studies are needed to evaluate the effects of the combined stresses of salinization and eutrophication on bacterial diversity, function and community assembly mechanisms, via seasonal field surveys and experiments that contain broader gradients of salinity and nutrient levels.

## 5. Conclusions

Our findings demonstrate that both aquatic bacterial species diversity and β-diversity decreased significantly under the combined stresses of salinity and nutrients in the semi-arid Inner Mongolia Plateau, China. Local environmental factors were the major determinant of BCC, with spatial factors also playing a significant role. Among the local environmental factors, salinity was the main driver of variations in BCC, while nutrient levels (especially nitrogen) were the main driver of variations in predicted BFC. Our results provide strong evidence that salinity-induced environmental filtering is a key determinant of bacterial community assembly in high-saline lake habitats. Stochastic processes dominated bacterial community assembly in freshwater and low-brackish lakes, contrasting with their minor role in the high-brackish habitat. Overall, our results highlighted the impact of climate warming-induced salinization, and intensive anthropogenic activity-induced eutrophication, on the loss of aquatic bacterial diversity, the shift of functional composition, as well as the relative importance of deterministic processes in community assembly, in this semi-arid area.

## Figures and Tables

**Table 1:** Characteristics of the elevation, climate parameters, water surface areas from the 1980s to 2018, mean water depth, salinity levels and trophic status of the five lakes in this study. The data come from references [23,24,25,29,30] and this study.

Lake	Elevation (m)	MAT (°C)	MAP (mm)	MAE (mm)	Water Surface Area (km^2^)			MWD (m)	Salinity Level	Trophic Status
					1980s	2000s	2018			
Hydrologically connected										
Durenor	1276	1.5	342	1630	2.2	2.1	1.9	3.5	freshwater	LE
Dalinor	1226	1.5	338	1632	227.8	222.6	190.0	7.5	low-brackish	HE
Ganggengnor	1243	1.6	360	1632	20.3	22.1	22.1	2.5	freshwater	HE
Hydrologically separated										
Chagannur	1013	1.7	278	2020	101.9	36.4	30.2	3.0	freshwater	HE
Daihai	1221	5.6	376	1670	137.4	87.4	58.1	7.0	high-brackish	ME

MAT, mean annual temperature; MAP, mean annual precipitation; MAE, mean annual evaporation; MWD, mean water depth in 2018; LE, light eutrophic; HE, hyper eutrophic; ME, middle eutrophic.

## Data Availability

Raw sequence data reported in this paper have been deposited in the Genome Sequence Archive in the BIG Data Center under accession number CRA002523. These data are publicly accessible at http://bigd.big.ac.cn/gsa.

## Supplementary Materials

The following are available online at https://www.mdpi.com/2076-2607/9/2/208/s1, Figure S1: Comparison of the 15 environmental parameters among the four sampling groups based on salinity gradients and ecosystem types. Figure S2: Rarefaction curves for the 40 samples analyzed using the Chao1 bias-corrected estimator. Figure S3: Bacterial operational taxonomic unit (OTU) composition and diversity. Figure S4: Relative abundance of the dominant genera in each sampling site ordered by ecosystem types (river vs. lake) and increasing salinity gradients. Figure S5: Mean proportions of the predicted functions across samples. Figure S6: Comparison of the predicted functions in the metabolization of energy and nitrogen. Figure S7: Spearman’s rank correlations between the Bray–Curtis similarity of bacterial communities and geographical distance. Table S1: Results of PERMANOVA and ANOSIM for comparison of bacterial community compositions and putative functional compositions.
