# Supplementary material for: Aquatic Bacterial Diversity, Community Composition and Assembly in the Semi-Arid Inner Mongolia Plateau: Combined Effects of Salinity and Nutrient Levels
Source: Microorganisms. 2021 Jan 20;9(2):208. doi: 10.3390/microorganisms9020208 (PMC7909399; doi:10.3390/microorganisms9020208)
Supplement: Supplementary file 1 [file microorganisms-09-00208-s001.pdf]

Supplementary Materials

Aquatic bacterial diversity, community composition and assembly in the semi-arid Inner Mongolia Plateau: combined effects of salinity and nutrient levels

Xiangming Tang [1,2,*], Guijuan Xie [1,2], Keqiang Shao [1], Wei Tian [1], Guang Gao [1,2], Boqiang Qin [1,2]

[1] *Taihu Laboratory for Lake Ecosystem Research, State Key Laboratory of Lake Science and Environment, Nanjing Institute of Geography and Limnology, Chinese Academy of Sciences, Nanjing 210008, China*

[2] *College of Resources and Environment, University of Chinese Academy of Sciences, Beijing 100049, China*

* Corresponding author: Xiangming Tang

xmtang@niglas.ac.cn; Tel. (+86) 25 86882242; Fax (+86) 25 57714759.

This Supporting Information including:

7 figures

1 table

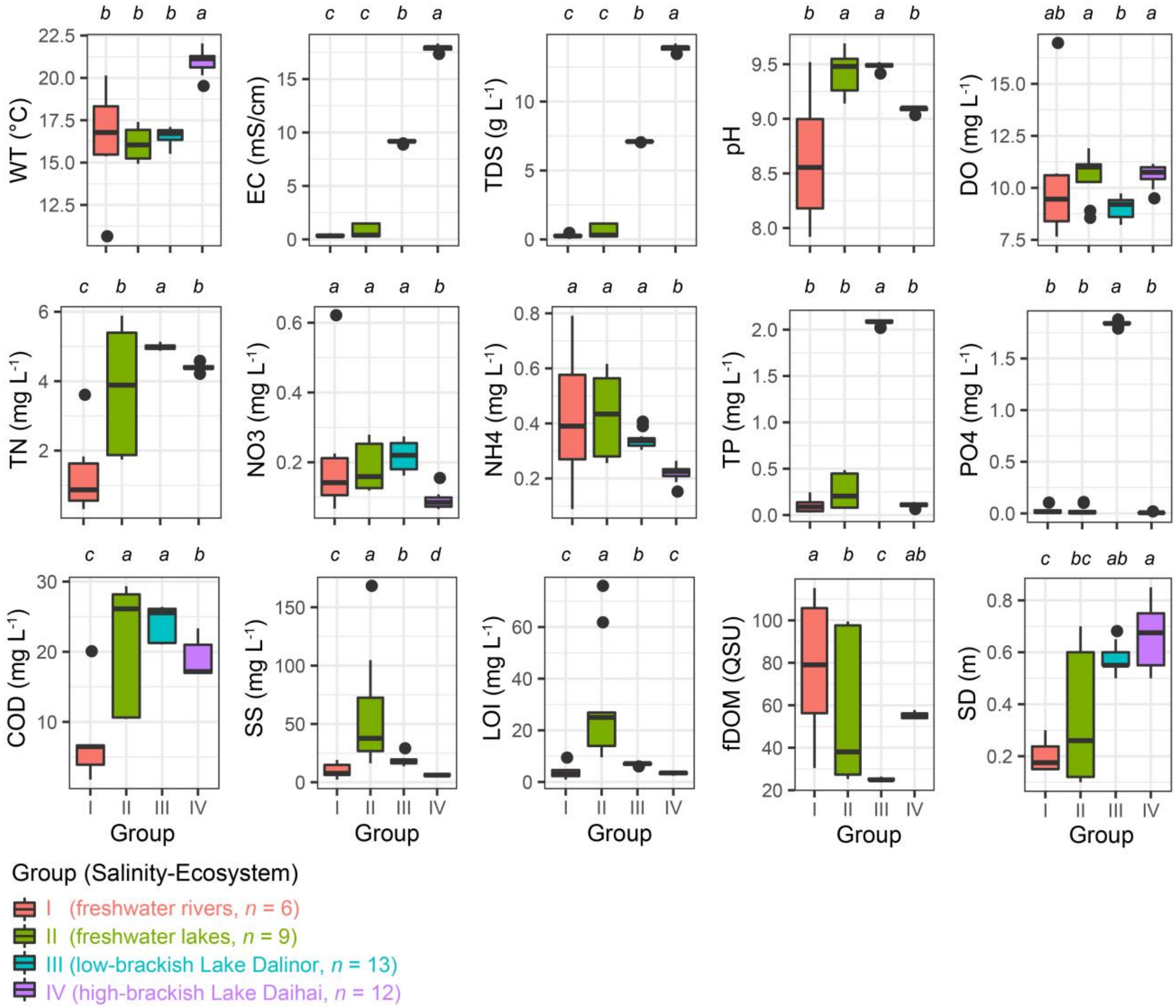

Figure S1. Comparison of the 15 environmental parameters among the four sampling groups based on salinity gradients and ecosystem types. In the box and whisker plots, horizontal bars in the box plots indicate median proportional values, and lower and upper edges of the boxes represent the first and third quartiles, respectively; upper and lower whiskers extend to data no more than 1.5 times the interquartile range from the upper edge and lower edge of the box, respectively. To examine differences among the four groups, Kruskal-Wallis tests were performed; different lower-case letters at the top of each panel indicate significant differences ($p < 0.05$) among groups. WT, water temperature; EC, electrical conductivity; TDS, total dissolved solids; DO, dissolved oxygen; TN, total nitrogen; NO3, nitrate; NH4, ammonia nitrogen; TP, total phosphorus; PO4, orthophosphate; COD, chemical oxygen demand; SS, suspended solids; LOI, loss on ignition; fDOM, fluorescent dissolved organic matter; SD, Secchi disk transparency. The unit of fDOM is quinine sulfate unit (QSU), where 1 QSU = 1 ppb quinine sulfate. Note that the SD in river samples is equal to their water depth.

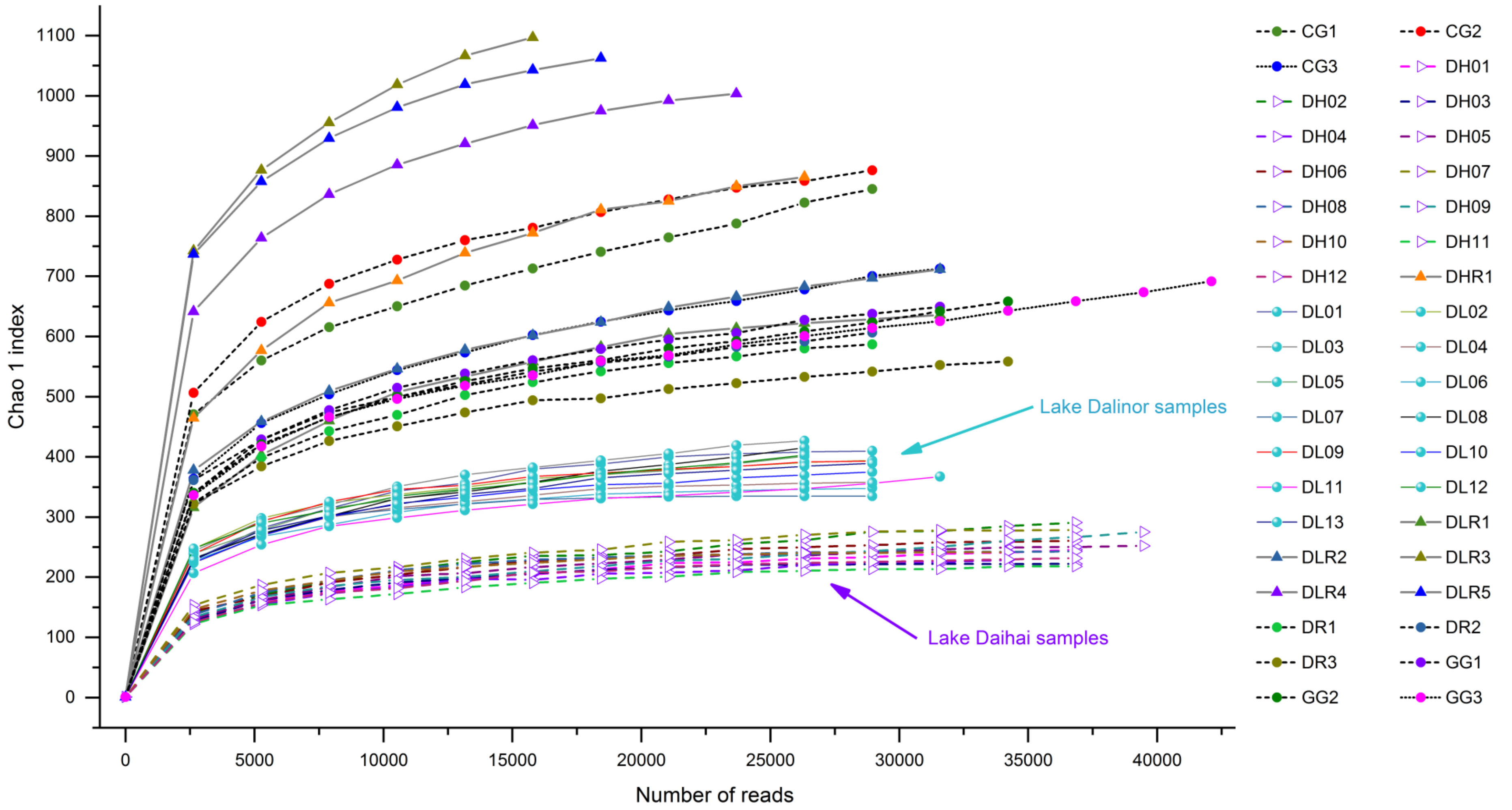

Figure S2. Rarefaction curves for the 40 samples analyzed using the Chao1 bias-corrected estimator. Sample locations: CG, Lake Chagannur (*n* = 3); DL, Lake Dalinor (*n* = 13); DLR, rivers flowing into Lake Dalinor (*n* = 5); DH, Lake Daihai (*n* = 12); DHR, river flowing into Lake Daihai (*n* = 1); DR, Lake Durenor (*n* = 3); GG, Lake Ganggengnor (*n* = 3).

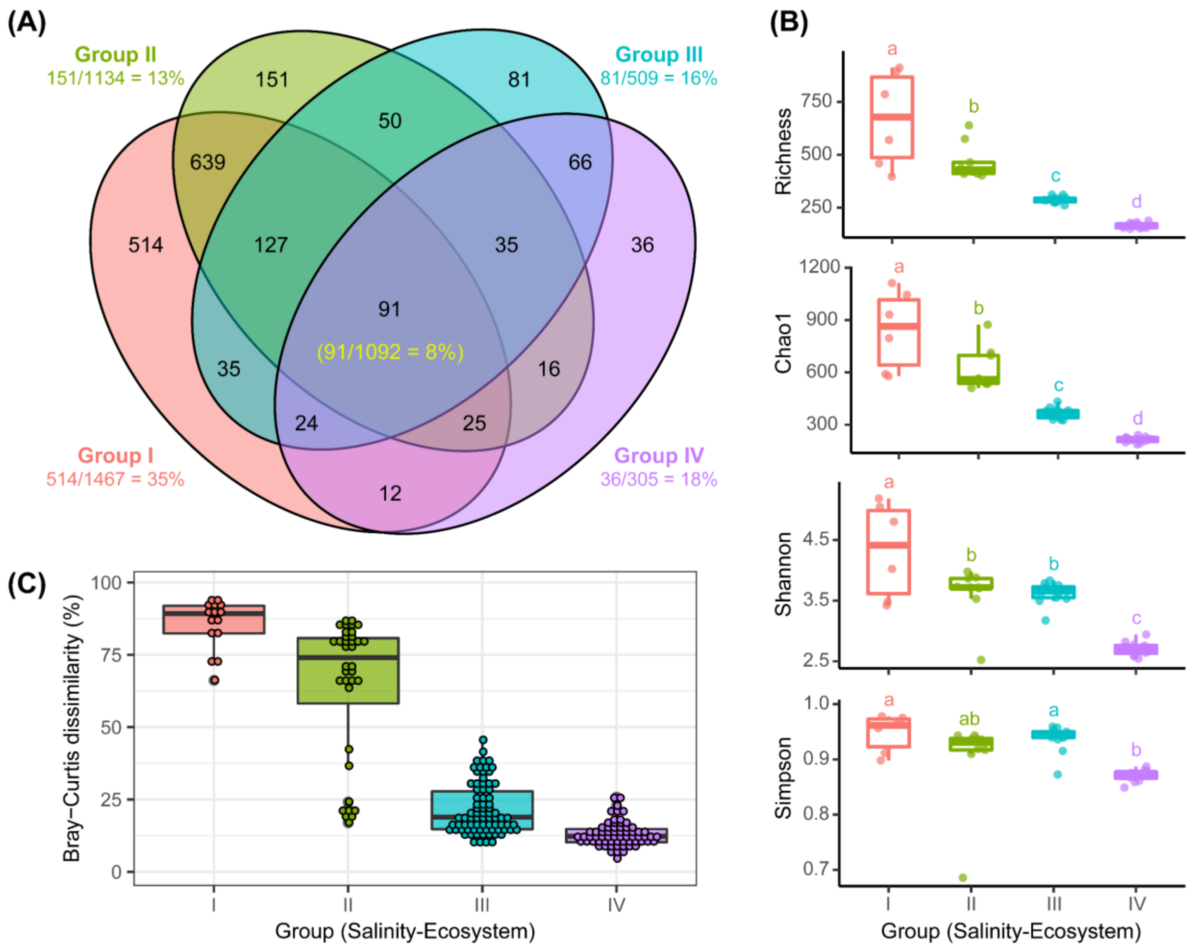

Figure S3. Bacterial operational taxonomic unit (OTU) composition and diversity among the four groups (Group I, freshwater rivers; Group II, freshwater lakes; Group III, low-brackish Lake Dalinor; and Group IV, high-brackish Lake Daihai). **(A)** Venn diagram showing bacterial OTU distribution. **(B)** Comparison of four bacterial α-diversity indexes using normalized sequencing depth based on the sample with the smallest sequencing effort (17,269 reads). Different lower-case letters indicate significant differences ($p < 0.05$) among groups. **(C)** Bray-Curtis dissimilarity between samples in different groups.

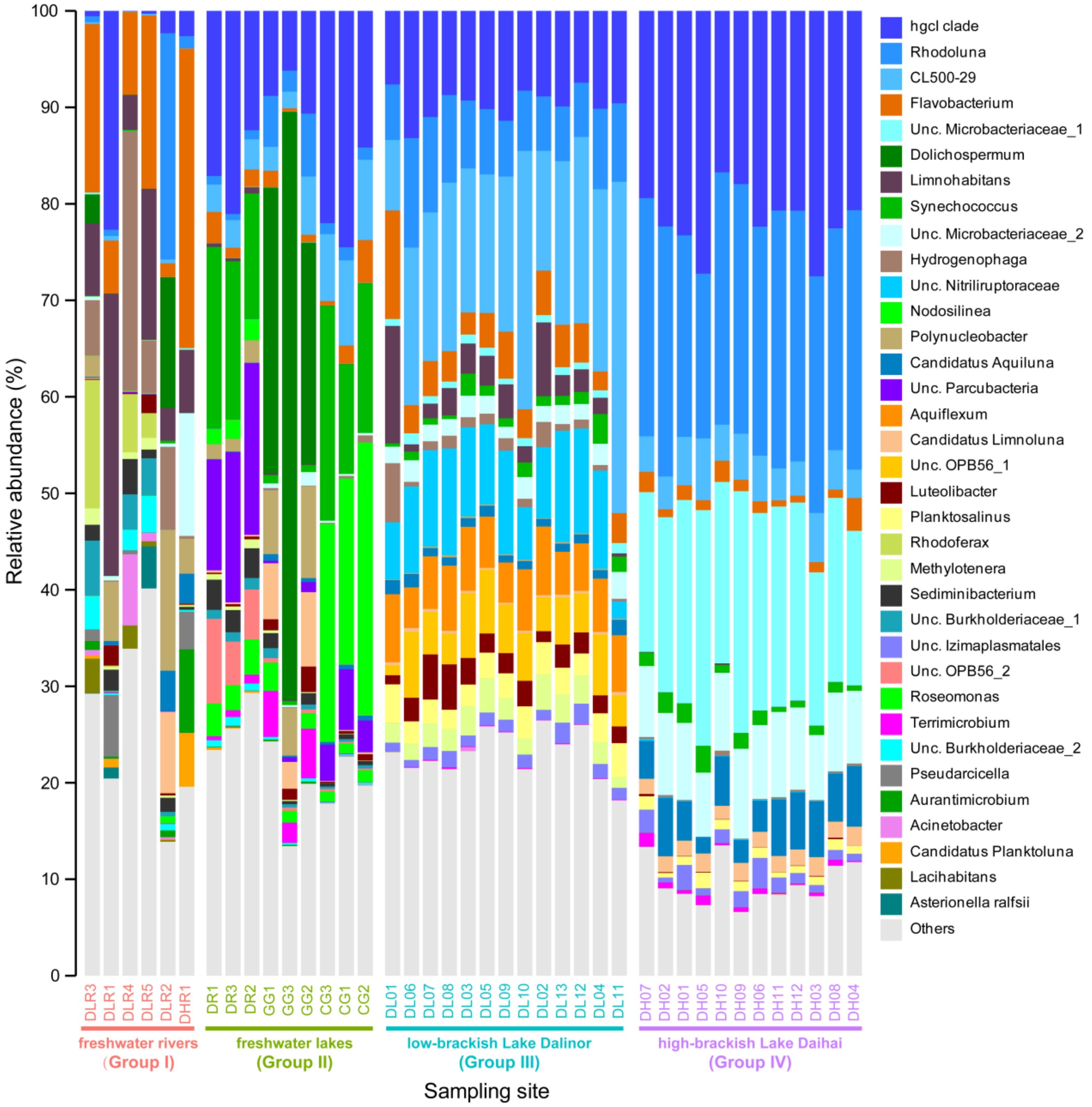

Figure S4. Relative abundance of the dominant genera in each sampling site ordered by ecosystem types (river vs lake) and increasing salinity gradients.

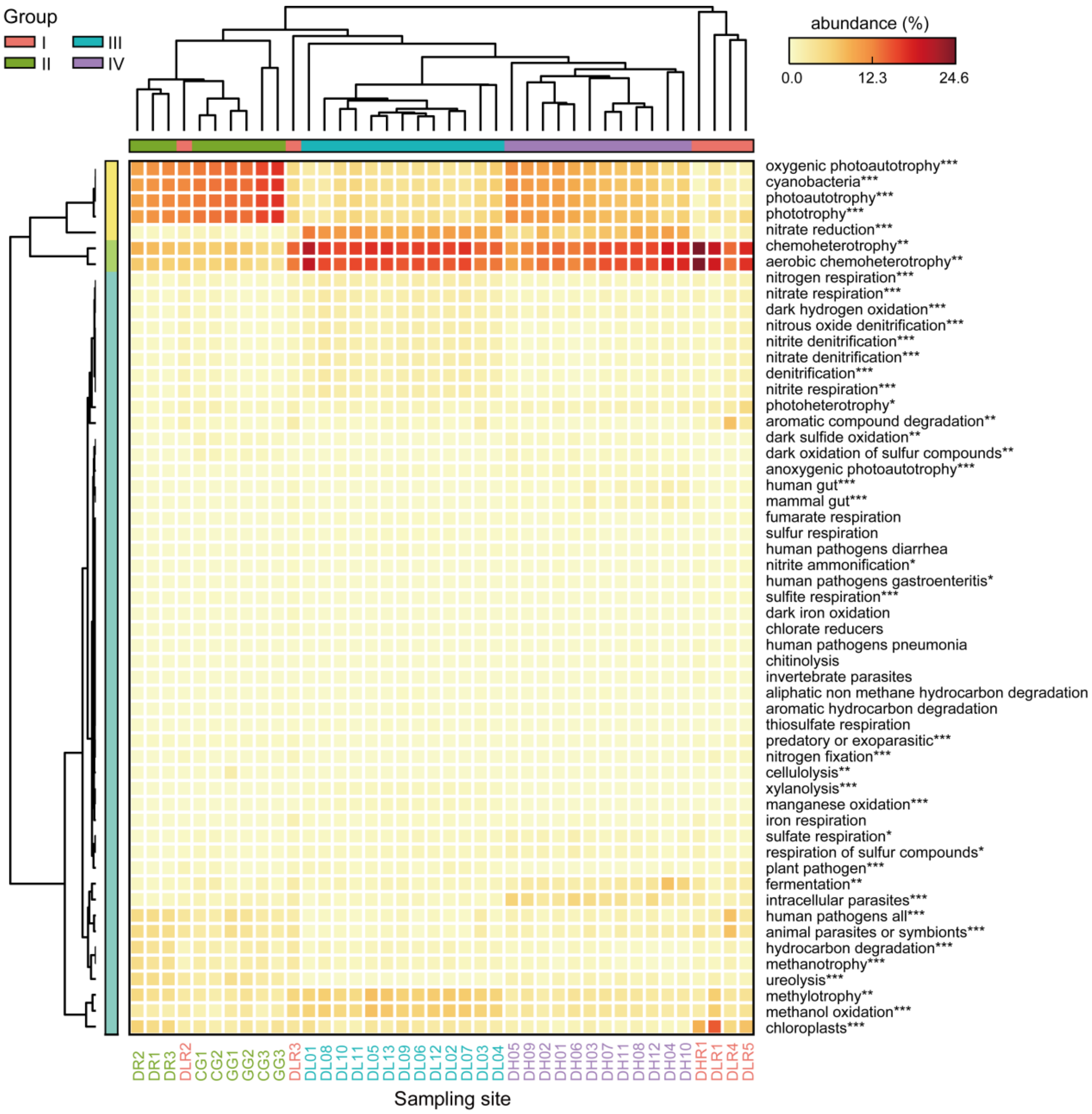

Figure S5. Mean proportions of the predicted functions across samples from freshwater rivers (Group I, $n = 6$), freshwater lakes (Group II, $n = 9$), saltwater Lake Dalinor (Group III, $n = 13$), and saltwater Lake Daihai (Group IV, $n = 12$). To test the significant difference in mean proportion of each function among different groups, the Kruskal-Wallis test with Bonferroni correction was performed: $*p < 0.05$, $**P < 0.01$, $***p < 0.001$.

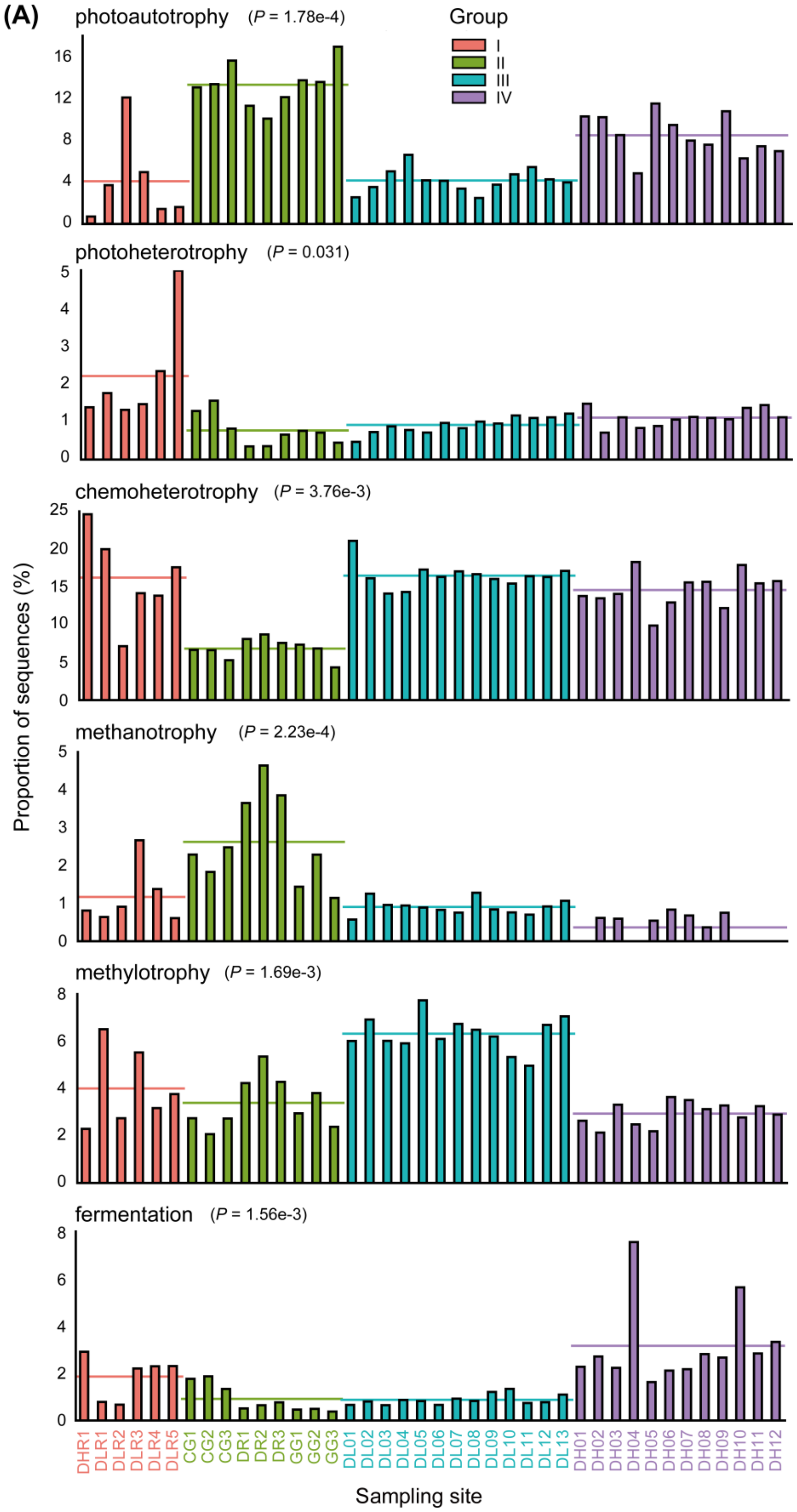
(A)
photoautotrophy (P = 1.78e-4)
Group
I
II
III
IV
photoheterotrophy (P = 0.031)
chemoheterotrophy (P = 3.76e-3)
methanotrophy (P = 2.23e-4)
methylotrophy (P = 1.69e-3)
fermentation (P = 1.56e-3)
Proportion of sequences (%)
DHR1 DLR1 DLR2 DLR3 DLR4 DLR5 CG1 CG2 CG3 DR1 DR2 DR3 GG1 GG2 GG3 DL01 DL02 DL03 DL04 DL05 DL06 DL07 DL08 DL09 DL10 DL11 DL12 DL13 DH01 DH02 DH03 DH04 DH05 DH06 DH07 DH08 DH09 DH10 DH11 DH12
Sampling site

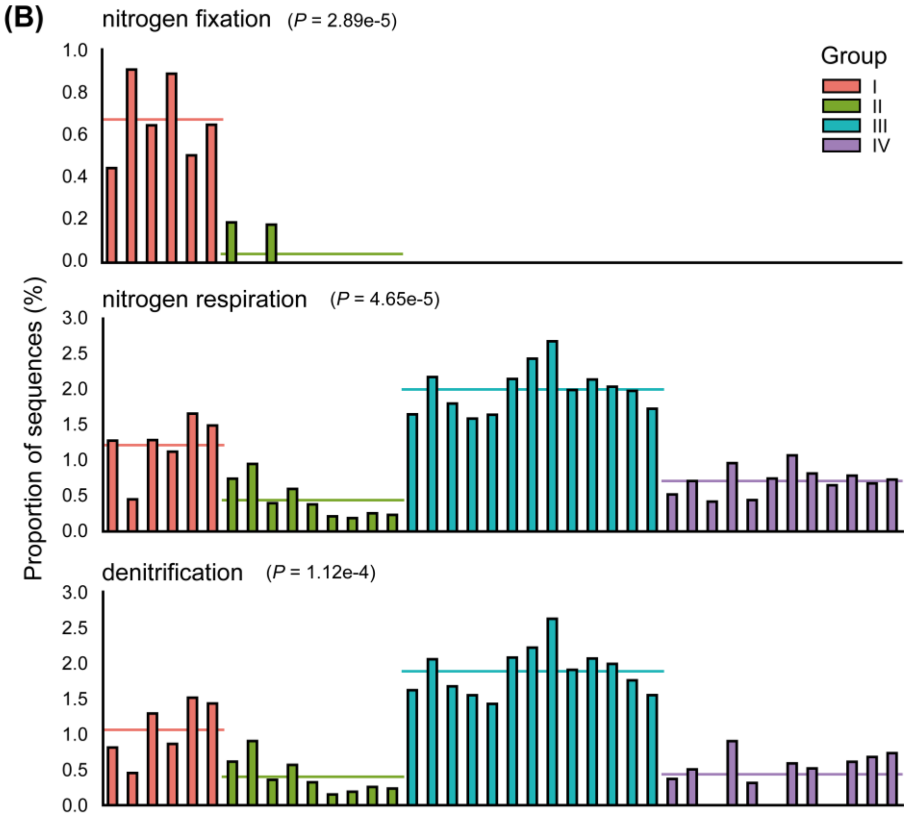

Figure S6. Comparison the predicted functions in the metabolization of (**A**) energy, and (**B**) nitrogen among samples from freshwater rivers (Group I, *n* = 6), freshwater lakes (Group II, *n* = 9), low-brackish Lake Dalinor (Group III, *n* = 13), and high-brackish Lake Daihai (Group IV, *n* = 12). To test the significance in mean proportion of each function among different groups, the Kruskal-Wallis test with Bonferroni correction was performed. Mean value for each group is shown by a separated horizontal line.

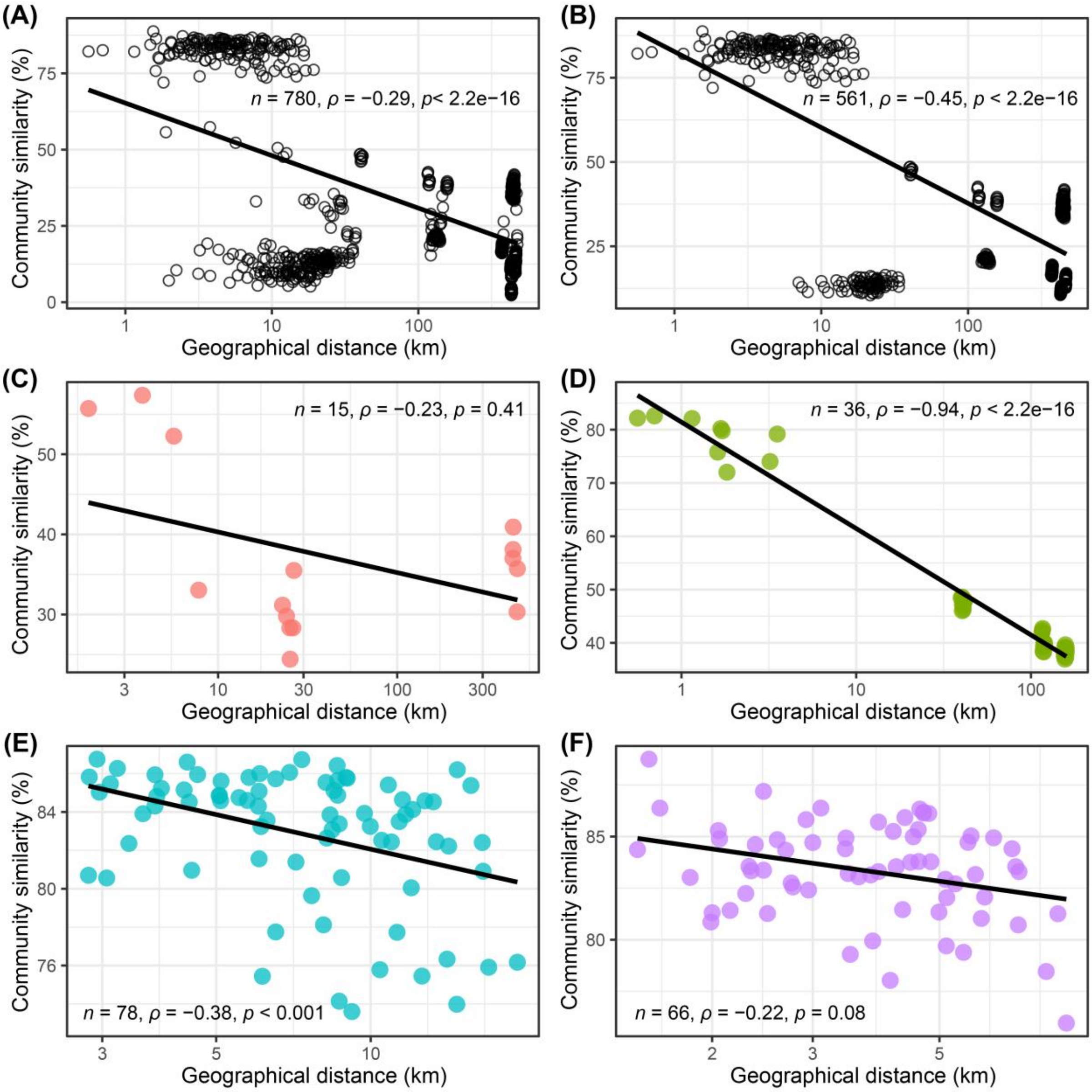

Figure S7. Spearman's rank correlations between the Bray–Curtis similarity of bacterial communities and geographical distance in **(A)** all samples, **(B)** all lake habitat samples, **(C)** freshwater river samples, **(D)** freshwater lake samples, **(E)** low-brackish Lake Dalinor, and **(F)** high-brackish Lake Daihai. Note: n, ρ, and p refer to the number of comparisons, rank correlations, and statistical significance, respectively.

Table S1. Results of permutational multivariate analysis of variance (PERMANOVA), and analysis of similarity (ANOSIM), for comparison of bacterial community compositions (BCCs) and putative functional compositions among samples from freshwater rivers (Group I, *n* = 6), freshwater lakes (Group II, *n* = 9), low-brackish Lake Dalinor (Group III, *n* = 13), and high-brackish Lake Daihai (Group IV, *n* = 12).

Groups	Bacterial community compositions (BCCs)				Putative functional composition			
	PERMANOVA		ANOSIM		PERMANOVA		ANOSIM	
	Pseudo-*t* statistic	*P*-value	Statistic *r*	*P*-value	Pseudo-*t* statistic	*P*-value	Statistic *r*	*P*-value
I vs II	2.6	0.0002	0.862	0.0002	4.7	0.0003	0.771	0.0004
I vs III	6.1	0.0001	1	0.0001	3.4	0.0001	0.857	0.0001
I vs IV	6.0	0.0002	1	0.0001	3.3	0.0003	0.819	0.0001
II vs III	7.0	0.0001	1	0.0001	13.5	0.0001	1	0.0001
II vs IV	6.9	0.0001	1	0.0001	6.5	0.0001	0.941	0.0001
III vs IV	12.6	0.0001	1	0.0001	7.1	0.0001	0.928	0.0001
